# Phosphorylation of astrocytic connexin43 by ERK1/2 impairs blood–brain barrier in acute cerebral ischemia

**DOI:** 10.1186/s13578-017-0170-6

**Published:** 2017-08-22

**Authors:** Wei Chen, Jiugeng Feng, Wusong Tong

**Affiliations:** 1 The People’s Hospital of Pu Dong New Area, 490 South Chuanhuan Road, Chuansha new town, Shanghai, 201299 People’s Republic of China; 2 Department of Neurosurgery, The First Affiliated Hospital of NanChang University, Yong Wai Zheng Street 17, Nanchang, 330006 China

**Keywords:** Connexin43, ERK, Phosphorylation, Blood–brain barrier, Ischemia

## Abstract

**Background:**

Connexins are a family of transmembrane proteins that form gap junctions, which are important for diffusion of cytosolic factors such as ions and second messenger signaling molecules. Our previous study has shown that Connexin40 (Cx40), one dominant connexin expressed in brain, was involved in brain injury. In this study, Cx43, another dominant connexin in brain, was investigated. Using bilateral common carotid artery occlusion-induced ischemia rat model, we tested the expression and phosphorylation level of Cx43 as well as heteromeric Cx40/Cx43 complex formation in brain after ischemia induction. We screened total 16 kinase inhibitors to identify the kinase for Cx43 phosphorylation and confirmed the result using siRNA targeting the specific kinase. Finally, we explored the role of the identified kinase in brain damage using in vivo rat model.

**Results:**

We discovered that phosphorylation of Cx43 increased after ischemia. The formation of Cx40/Cx43 heteromeric complex on membrane also increased. Inhibition of ERK activity resulted in inhibition of Cx43 phosphorylation on astrocytes. In in vivo model, application of ERK inhibitor and siRNA prevented brain damage and protected blood–brain barrier integrity in rat.

**Conclusion:**

Our study provides evidence that Cx43 phosphorylation by ERK is implicated in ischemia induced brain damage.

## Background

A stroke is when poor blood flow to the brain results in cell death. The two major mechanisms causing brain damages in stroke are ischemia and hemorrhage [1]. In ischemic stroke, which represents about 80% of total [2], the integrity of blood–brain barrier (BBB) is severely impaired following reduced or blocked circulating blood. Physiologically, blood–brain-barrier exemplifies a diffusion barrier, which consists of an interdependent network to segregate the central nervous system (CNS) from the systemic circulation, and rigorously regulates para-cellular permeability [3].

The restrictive angioarchitecture at the BBB reduces paracellular diffusion, while minimal vesicle transport activity in brain endothelial cells limits transcellular transport. The properties of the BBB are primarily determined by junctional complexes between the cerebral endothelial cells, comprised of tight, adherens and gap junctions [4].

Gap junctions (GJs) are composed of thousands of intercellular channels permeable to cytosolic factors, such as ions and second messenger signaling molecules [5]. Gap junctions are formed by members of the connexin (Cx) family. Connexins are transmembrane proteins. Cxs oligomerize to from connexons (hemichannels) which are defined as homomeric when the comprising Cxs are identical or heteromeric when two or more Cxs comprise the connexon. Connexins in adjacent cells join the extracellular space to from the functional intercellular channel, which is defined as homotypic when the Cx composition of the contribution is identical or heterotypic when different. Connexins exhibit tissue-specific expression and brain cells express Cx37, Cx40 and Cx43 [4].

Both Cx40 and have been shown to be associated with brain pathophysiology. For example, Deng et al. reported that inhibition of Cx43 by leptin contributes leptin’s neuroprotective activity against brain ischemic injury [6]. Merier and colleagues detected the differences in expression of Cx43 in response to brain injury, and they also found overexpression or deletion of Cx43 was associated with the pathophysiological outcome [7]. Our previous data showed that in a traumatic brain injury (TBI) mice model, Cx40 protein expression was up-regulated as early as 6 h post brain injury. Ginsenoside Rb1 (GS-Rb1) exerted neuroprotective activity against TBI by down-regulating Cx40 expression, which depended on activation of ERK [8, 9].

The majority of connexins including Cx40 and Cx43 have been shown to be phosphoproteins. Changes in connexin phosphorylation status are closely associated with changes in GJ assembly, stability and channel properties [10]. Cx43 is phosphorylated primarily on multiple serine residues by several protein kinases including protein kinase C (PKC) [11], MAP kinase [12], and pp60^src^ kinase [13]. Phosphorylation of Cx43 has been reported in different tissues. Immunohistochemical analysis of mouse heart proved that the phosphorylation of Cx43 on serine 365, S325/S328/S330 occurs at intercalated disc [14]. Remo et al. [15] have shown that Cx43 phosphorylation status at these sites is necessary for adaptive response in cardiac tissue to change in physiologic parameters. Murry and colleagues studied the changes in Cx43 phosphorylation and localization during cardiac ischemia and elucidated the critical role of Cx43 in the phenomenon of preconditioning [16].

In brain, Cx43 is the dominant gap junction protein expressed in astrocytes. Many diverse functions have been attributed to Cx43 and gap junctional communication. Although Cx43 has been shown to increase in human brain in ischemia [17] and in the hippocampus of patients with epilepsy [18], the phosphorylation of Cx43 has not been analyzed in detail. Here we demonstrated that in the rat model of acute cerebral ischemia, the phosphorylation of Cx43 increased after ischemia, correlated to the increased phosphorylated Cx43/Cx40 complex on membrane. Cx43 was phosphorylated by ERK. Knocking down ERK by siRNA or inhibiting ERK activity by ERK inhibitor blocked the phosphorylation of Cx43, protected blood–brain barrier integrity and prevented brain damage in ischemia.

## Methods

### Animals

This study was carried out in strict accordance with the recommendations in the Guide for the Care and Use of Laboratory Animals of the National Institutes of Health. The protocol was approved by the Committee on the Ethics of Animal Experiment of The People’s Hospital of Pu Dong New Area. All surgeries were performed under sodium pentobarbital anesthesia, and all efforts were made to minimize suffering. Wistar rats were purchased from SLAC laboratory animal (Shanghai, China). Rats were caged in fully ventilated room and maintained in 12:12 h light and dark cycle. They had free access to a standard chow diet and water.

### Experimental design

Wistar rats were randomly divided into two groups. In Group I (Sham control) cerebral ischemia was not induced. Group II cerebral ischemia was induced. At 2, 4, 6 and 8 h post cerebral ischemia induction, 8 rats from group II were taken out for analysis.

### Induction of cerebral ischemia

Surgical technique for induction of cerebral ischemia was adapted from the method of Iwasaki et al. [19]. Briefly, rates were anesthetized by giving sodium pentobarbital (75 mg/kg, intraperitoneal). A midline incision was made to expose both the right and the left common carotid arteries. Each carotid artery was freed from its adventitial sheath and vagus nerve, which was carefully separated and maintained. Cerebral ischemia was induced by clamping both the arteries using microvascular clips. Throughout the surgical procedure, the body temperature was maintained at 37 °C using a surgery plate connected to a temperature stabilizer. In the sham control group, both the carotid arteries were surgically exposed, but occlusions were not made. The abrupt drop in cortical perfusion (<25% of the baseline) was monitored by the laser Doppler flowmetry (PRIMED Inc. Stockholm, Sweden). After 30 min of bilateral common carotid artery occlusion, the microvascular clips were removed to permit for reperfusion (>75% of the baseline).

### RT-PCR

The tissue sample was collected from the ipsilateral cortex of the cerebral ischemia animal 2, 4, 6 and 8 h post cerebral ischemia induction or from sham animal. The total RNAs were extracted with use of RNeasy Mini Kit (Qiagen, CA, USA). Reverse transcription was performed using a reverse transcription kit (Applied Biosystem) as recently described. Real time quantitative PCR reactions were set up in triplicate wit Ssofact Master Mix (Biorad, CA, USA) and run on a LightCycler 480 (Roche, Penzberg, Upper Bavaria, Germany). The following primers were used in the current study: Cx40: (Fwd: GAAAGAGGTGAACGGGAAGA, Rev: GCCACAGCCATCATAAAGACA); Cx43: (Fwd: TGCTTGGGATAGCTGGGCGGA, Rev: TGGGGGCAGAGAGAGAAAGCCC); GAPDH: (Fwd: CAAGTTCAACGGCACAGTCAAG, Rev: ACATACTCAGCACCAGCATCAC).

### Culture of cortical astrocytes

Isolation and culture of rat crotical astrocytes were followed the protocol described previously [20]. Briefly, Wistar rats had their cerebral cortices aseptically dissected and meninges removed. During the dissection, the cortices were kept in HBSS (Hank’s Balanced Salt Solution) containing 0.05% trypsin and 0.003% DNase at 37 °C for 15 min. The tissue was then mechanically dissociated for 15 min using a Pasteur pipette and centrifuged at 400*g* for 5 min. The pellet was re-suspended in a solution of HBSS containing 40 U papain/ml, 0.02% cysteine and 0.003% DNase and again gently mechanically dissociated for 15 min. After another centrifugation step (400*g*, 5 min), the cells were resuspended in HBSS containing only DNase (0.003%) and left for decantation for 30–40 min. The supernatant was collected and centrifuged for 7 min (400*g*). The cells from supernatant were resuspended in DMEM/F12 [10% fetal bovine serum (FBS), 15 mM HEPES, 14.3 mM NaHCO_3_, 1% fungizone and 0.04% gentamicin], plated in 6- or 24-well plates pre-coated with poly-l-lysine and cultured at 37 °C in a 5% CO_2_ incubator. Medium was changed every 2 days.

### Brain water content

Brain water content was measured using Hatashita’s wet-dry method. Briefly, rats were sacrificed by cervical dislocation and their brains were immediately removed and placed onto a frozen plate and then weighed to determine wet weight. Next, brains were dried in a desiccating oven at 110 °C for 24 h and weighed again to determine the dry weight. Brain water content was calculated using the following formula: brain water content (%) = (wet weight − dry weight) × 100/wet weight.

### Assessment of Evans blue extravasation

The assay of Evans blue (EB) leakage was used to evaluate blood–brain-barrier (BBB) disruption following the experimental stroke, as described previously [21]. Briefly, 2% EB (4 ml/kg) was injected via the tail vein at 6 h after reperfusion. At 2 h after EB injection, rats were perfused under anesthesia. Tissue samples were collected from the cortex and striatum, which were then homogenized by 50% trichloracetic acid and centrifuged. The absorbance of supernatants was measured at 620 nm with infinite M200 PRO (TECAN, Austria).

### Surface biotinylation of slices

The protocol of brains slices making and the surface biotinylation was followed as previously described [22]. Briefly, 2, 4, 6 and 8 h post cerebral ischemia, brains were dissected and placed on to a plate with ice-cold standard physiological solution (ASCF). The brains were cut into 350 µM slices. Then the slices were biotinylated in the biotinylation reagent solution of 100 µM NHS-SS-biotin (Sigma, St. Louis, MO, USA) in ASCF. After 45 min of incubation, the slices were washed twice with ASCF buffer containing 1 µM lysine (Sigma, USA) to block all reactive NHS-SS-biotin in excess.

### Immunoprecipitation, western blot and dot blot assay

For biotinylated slices, samples were collected and transferred to an Eppendorf tube, washed twice with ice-cold ASCF containing 1 µM Lys and then lysis in RIPA lysis buffer containing 1% protease inhibitor cocktail (Santa cruz, Dallas, TX, USA). A total of 30 µg of protein were loaded onto a 12% SDS-PAGE gel. After transfer, membranes were blocked by 5% non-fat milk and incubated with rabbit anti-Cx40, goat anti-Cx43 (Santa Cruz, USA), rabbit anti-p-Cx43 (Abcam, Cambridge, MA, USA) for overnight at 4 °C. Next day, corresponding HRP-conjugated secondary antibodies were incubated. After washing, the membranes were finally carried out with SuperSignal1 West Pico Chemiluminenscent Substrate Kit (Pierce Biotechnology, USA). For some experiment, proteins were eluted from the streptavidin bead by using 0.2 M Glycine (pH 2.7) and immediately neutralize by Tris buffer (pH 8.0). Immunoprecipitation using anti-Cx40 antibody was carried out following protocol described previously [23]. The dot blot assay was carried out following the standard protocol from Abcam.

### siRNA transfection

siRNA against ERK1/2 was purchased from Cell Signaling Technology (#6560). siRNA was transfected into astrocytes using Lipofectamine 2000 (Invitrogen, Carlsbad, CA, USA) following the manufacture’s protocol. siRNA was transfected in vivo using Invivofectamine 3.0 (Invitrogen, USA) 48 h prior to cerebral ischemia.

### Kinase inhibitor

Totally 16 kinase inhibitors (Table 1) were purchased from selleck. These inhibitors were added to astrocytes directly. For the in vivo experiment, 1, 5 and 10 µg/g (brain) of ERK inhibitor SCH772984 was injected into cerebral ventricle 2 h prior to cerebral ischemia following protocol described previously [24].

**Table 1:** Reagents used in this study

#	Inhibitor	Target	Working concentration
1	DMSO	Control	–
2	Wortmannin	PI3K	1 µM
3	Flavopiridol	CDK	40 nM
4	Erlotinib	EGFR	1 µM
5	Ruxolitinib	JAK	1 µM
6	MK-2206	AKT	1 µM
7	AICAR	AMPK	2 mM
8	Y-27632	ROCK	10 µM
9	SCH772984	ERK	1 µM
10	NSC23766	Rho	10 µM
11	Enzastaurin	PKC	1 µM
12	SP600125	JNK	10 µM
13	GSK2334470	PDK	1 µM
14	IPA-3	PAK	20 µM
15	Axitinib	VEGFR	1 µM
16	PHA-665752	c-Met	10 µM

### Statistical analysis

Statistical analysis was performed using SPSS 16.0 software. Data were presented as mean ± standard error of the mean (SEM). Two-way analysis of variance (ANOVA) followed by Tukey’s post hoc test was used to determine between-group differences. Statistical difference was considered to be significant only if *p* < *0.05*.

## Results

### Bilateral common carotid artery occlusion up-regulated Cx40 phosphorylation

**Fig. 1:**
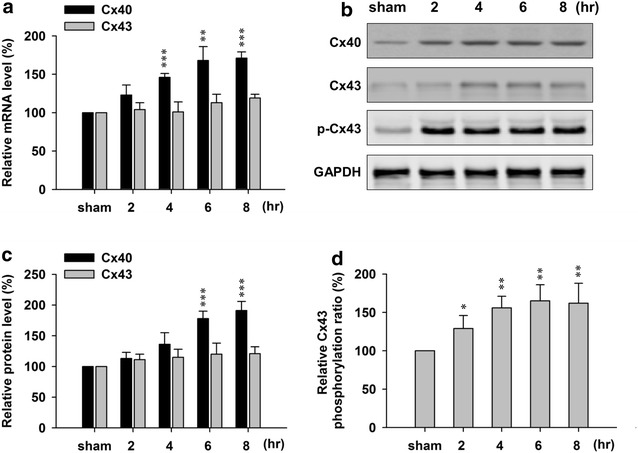
Effects of bilateral common carotid artery occlusion-induced ischemia on Cx40 and Cx43 mRNA and protein levels in the brain. **a** qPCR results of Cx40 and Cx43 at sham (0), 2, 4, 6, 8 h after occlusion. **b** Western blot results of Cx40 and Cx43 protein level changes at sham (0), 2, 4, 6, 8 h after occlusion. **c** Normalized protein expressions of Cx40 and Cx43, and **d** Normalized phosphorylation ratio of Cx43 at sham (0), 2, 4, 6, 8 h after occlusion. Data is presented as mean ± SEM. *p < 0.05, **p < 0.01 and ***p < 0.001

Consistent with previous data [8], we found that bilateral common carotid artery occlusion induced brain ischemia up-regulated the expression of Cx40 in both mRNA (Fig. 1a) and protein (Fig. 1b, c) levels after ischemia induction. There was significantly increased Cx40 mRNA as early as 4 h after and significantly increased protein level as early as 6 h. Although there was no significant difference of Cx43 mRNA or protein level after ischemia induction, we observed concomitant up-regulation of Cx43 phosphorylation as early as 2 h post (Fig. 1b, d). These results suggested that phosphorylation of Cx43 may be involved in bilateral common carotid artery occlusion induced brain damage.

### Ischemia induced increased Cx40/Cx43 complex on membrane

**Fig. 2:**
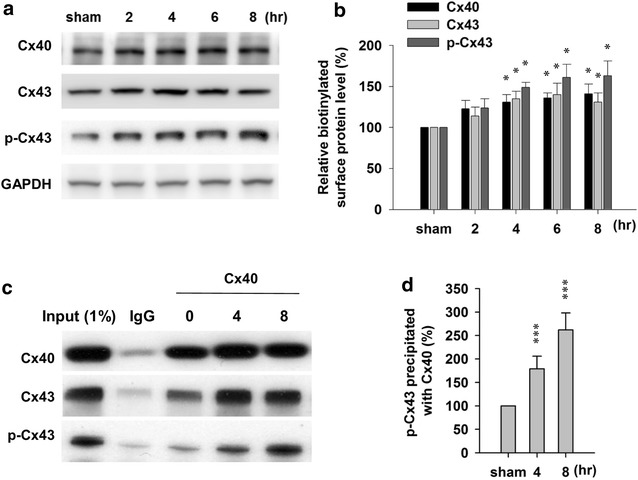
Ischemia-induced modifications of Cx40/Cx43 complex. **a** Western blot tests of Cx40, Cx43 and p-Cx43 from biotinylated membrane which was eluted from Dynabeads^®^ MyOne™ Streptavidin C1 beads. GAPDH in the total lysate was used as control. **b** Statistic results after normalization. **c** Sample images and statistic results (**d**) of Cx40, Cx43 and p-Cx43 immunoprecipitated with Cx40 antibody. Data is presented as mean ± SEM. *p < 0.05, **p < 0.01 and ***p < 0.001

Connexins are transmembrane proteins and they form the connexons on cell membrane. Next we investigated the Cx40/Cx43 complex on cell membrane after ischemia induction. As shown in Fig. 2a, b, we can observe the significantly increased amount of both Cx40 and Cx43 on the membrane as early as 4 h post ischemia induction. It has been reported that Cx43 and Cx40 can from heteromeric gap junction channels [25, 26]. More interestingly, by using Cx40 antibody, we detected increased Cx43 and phosphorylated Cx43 co-immunoprecipitated with Cx40 after ischemia induction (Fig. 2c, d). These results not only confirmed the formation of Cx43/Cx40 heteromeric channels but also suggested the involvement of Cx43/Cx40 and phosphorylated-Cx43/Cx40 heteromeric channels in brain damage during ischemia.

### ERK1/2 signaling regulated Cx43 phosphorylation

**Fig. 3:**
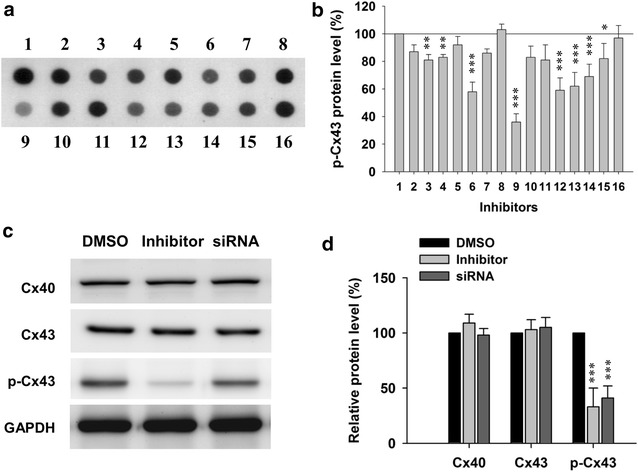
ERK1/2 signaling regulates phosphorylation of Cx43. **a** Sample image and **b** statistical analysis of Cx43 phosphorylation in astrocytes after application of 16 kinase inhibitors. **c**, **d** ERK inhibitor SCH772984 and siRNA effectively reduced Cx43 phosphorylation level without affecting Cx40 and Cx43 total protein levels in cultured astrocytes. Data is presented as mean ± SEM. *p < 0.05, **p < 0.01 and ***p < 0.001

Cx43 has been reported to be phosphorylated by several kinases, including PKC, p38 MAPK and ERK1/2 [14]. To identify the possible kinase/kinase signal pathway which is responsible for Cx43 phosphorylation in brain during ischemia, we screened total 16 kinase or kinase signaling pathway inhibitors for their inhibition activity of Cx43 phosphorylation on astrocytes, which are the dominant cells expressing high level of Cx43 in brain [17]. As shown in Fig. 3a, b, 8 out of 16 kinase inhibitors can significantly inhibit the phosphorylation of Cx43 in astrocytes on the dot blot assay. Among the 9 inhibitors, SCH772984, which was known as ERK inhibitor, was the most effective. The inhibition activity of SCH772984 on Cx43 phosphorylation was confirmed on western blot. As shown in Fig. 3c, d, SCH772982 treatment significantly decreased the Cx43 phosphorylation level while did not change the total protein level. These results suggested that ERK1/2 was responsible for Cx43 phosphorylation in astrocytes. This conclusion was confirmed by siRNA treatment. As shown in Fig. 3c, d, siRNA treatment to knock down ERK also decreased the Cx43 phosphorylation level.

### ERK1/2 was involved in brain damage during ischemia

**Fig. 4:**
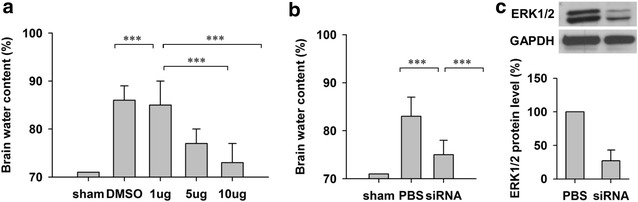
In vivo administration of ERK inhibitor SCH772984 or siRNA prevents ischemia-induced brain infarction. **a** Different dosage of SCH772984 treatment (1, 5, 10 µg/g brain) attenuate brain edema at 8 h after bilateral common carotid artery occlusion. **b** In vivo transfection of ERK1/2 siRNA attenuates brain edema at 8 h after bilateral common carotid artery occlusion. **c** Western blotting results of the ERK1/2 protein level reduction at 48 h after siRNA transfection. Data is presented as mean ± SEM. *p < 0.05, **p < 0.01 and ***p < 0.001

Brain injuries after ischemia induction was demonstrated by increased brain infarction volume and brain water content [8]. We already showed that ischemia was associated with increased Cx43 phosphorylation (Fig. 1). As inhibiting ERK1/2 resulted in decreased Cx43 phosphorylation, we next investigated the effects of ERK1/2 inhibition on brain damage. As shown in Fig. 4a, rats treated with SCH772984 showed decreased brain water content in a dose dependent manner. Rats treated with 5 or 10 µg SCH772984 demonstrated significant decreased brain water content compared with mock treated group. Similarly, ERK siRNA treated rat s also showed significant decreased brain water content (Fig. 4b). The knocking-down efficiency of the above siRNA in the brain was verified by Western-blot at 48 h after siRNA transfection (Fig. 4c). These results suggested that ERK participated in brain damage during ischemia.

### Inhibition of ERK1/2 protected blood–brain barrier integrity

**Fig. 5:**
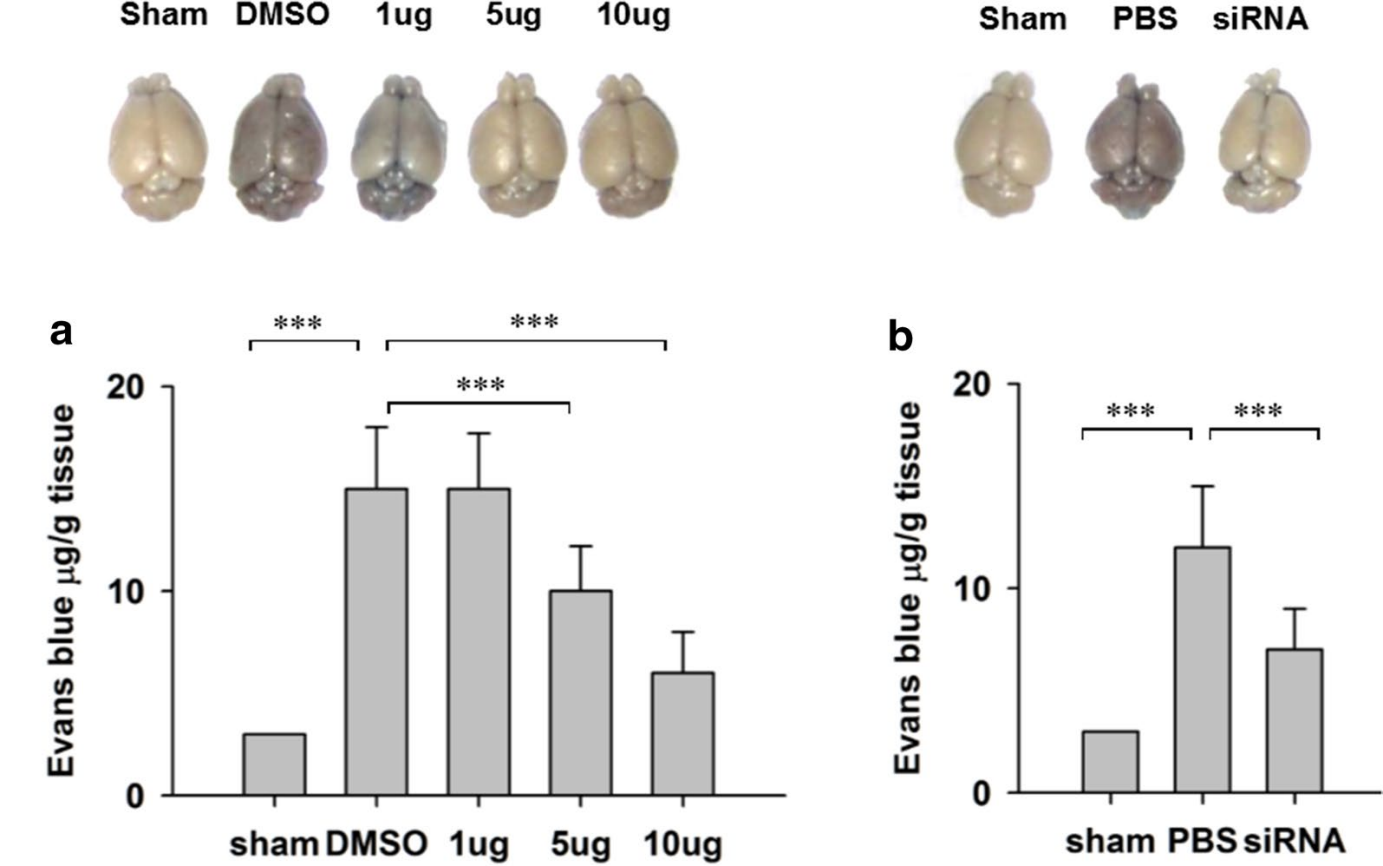
In vivo administration of ERK inhibitor or siRNA defends ischemia-induced impairment in blood–brain barrier integrity. **a** SCH772984 treatment protects blood–brain barrier integrity after bilateral common carotid artery occlusion. **b** siRNA against ERK1/2 treatment protects the blood–brain barrier integrity after ischemia induction. Data is presented as mean ± SEM. *p < 0.05, **p < 0.01 and ***p < 0.001

The integrity of blood–brain barrier was impaired during ischemia [8]. As inhibition of ERK impaired brain damage in ischemia, we next explored the effect of ERK inhibition on blood–brain barrier integrity. 8 h after bilateral common carotid occlusion, EB leakage into the ipsilateral hemispheres was significantly decreased in both ERK inhibitor treated rat group (Fig. 5a) and ERK siRNA treated rat group (Fig. 5b). These results indicated that ERK activity contributed to blood–brain barrier damage during ischemia and inhibition of ERK activity impaired the damage.

## Discussion

Connexin proteins are integral components that form hemichannels or gap junction complexes which allow for exchange of ions and small molecules [5]. Gap junctions play important role in brain damage in vitro as well in vivo. Several studies have showed that gap junctions contribute to the brain damage [27]. The first evidence supporting the role of gap junctions in mediating spread of damage in CNS was that octanol, a gap junction blocker, reduced infarct volume after focal ischemia [28]. A recent study showed that blockage of connexin hemichannel was neuroprotective after global cerebral ischemia in near-term fetal sheep [29]. In the rat brain, down-regulation of specific connexins reduced neuronal cell death in a global ischemia model [30]. Our previous data have shown that the expression of connexin40, one of the major connexins expressed in brain, was upregulated in traumatic brain injury. Application of Ginsenoside Rb1 protected brain against traumatic brain injury by down-regulating the Cx40 expression [8, 9]. These results indicated the role of Cx40 in brain damage.

Connexins have been shown to be phosphoproteins. Phosphorylation has been implicated in the regulation of a broad variety of connexin processes [31]. The role of connexin43 phosphorylation/de-phosphorylation has been studied. Cx43 in ventricle is highly phosphorylated and the phosphorylation site are S365 and S325/S328/S330 [14]. The phosphorylation on these sites is linked to gap junction remodeling and arrhythmic susceptibility. Cx43 phosphorylation has also been implicated in affecting vessel biology. For example in resistance vessels, Cx43 phosphorylation regulates the electrical and dye coupling between vascular smooth muscle cells and endothelial cells [14]. Beardslee and the colleagues reported that ischemia causes de-phosphorylation of connexin43 in cardiac muscle [32]. Using an in vitro astrocytes culture model, Li et al. [33] reported that astrocytes contained predominantly phosphorylated Cx43 and these underwent dephosphorylation after hypoxia. In current study, we found increased phosphorylation of Cx43 in the brain after ischemia. Our result was different from previous reports. This can be explained by the difference of the model used and the difference of organs analyzed. Our data was from the brain of an in vivo rat model after bilateral common carotid artery occlusion, which could be more relevant. Thus, this correlation suggested a potential role of phosphorylated Cx43 in ischemia induced brain damage, which need to be further investigated. As the phosphorspecific antibodies are available, it should be very useful to identify the phosphorylation site of Cx43 in brain during ischemia, which will give more information for the function of Cx43 phosphorylation during ischemia.

Connexins are transmembrane proteins and they form the connexons on cell membrane. Six connexins oligomerize to form a hemichannel, or connexon, and two connexons from adjacent cells dock to form a functional gap junction channel. Connexons composed of only on connexin isoform are termed homomeric, whereas connexons formed from more than one connexin isoform are labeled heteromeric [25]. In this study, our immunoprecipitation data indicates that Cx40 and Cx43, or Cx40 and phosphorylated Cx43 can form heteromeric connexons, which is already reported previously [25]. We also found the increased Cx40/Cx43 and Cx40/phosphorylated Cx43 complex on membrane in ischemia. It has been reported that the heteromeric channels are not consistent with homomeric channels. The heteromeric channels display a strongly asymmetric voltage-dependent gating response. Thus, it is possible that the increased Cx40/Cx43 and Cx40/phosphorylated Cx43 heteromeric channels on the membrane result in gap junction changes after ischemia induction and these changes contribute to brain damage.

Cx43 is phosphorylated by several known and unknown protein kinases. The known proteins include protein kinase C (PKC), MAP kinase and the pp60^src^ kinase. Activation of the EGF and PDGF receptor tyrosine kinases by ligand binding also results in a marked increase in the phosphorylation of Cx43 [10]. We identified that inhibition of ERK by inhibitor SCH772984 or siRNA treatment to knock down ERK can inhibit Cx43 phosphorylation, suggesting ERK is the upstream kinase for Cx43 phosphorylation. 7 other inhibitors targeting CDK, EGFR, AKT, JNK, PDK, PAK and VEGFR also shows inhibitory activity of Cx43 phosphorylation, but with less efficiency when compared to ERK inhibitor. Among these 7 targets, EFGR is an upstream component of the MAPK/ERK pathway while other molecules participate in other signal transduction pathways. It is possible that besides EGFR/ERK signal pathway, Cx43 is also the substrate of these kinases. It is also possible that the inhibitory effect on Cx43 phosphorylation of these inhibitors come from off-target side effects. All these results indicate a potential role of ERK in brain damage, possibly through phosphorylation of Cx43. Based on these, we continue to test using in vivo rat model and prove that inhibiting ERK activity prevent brain damage and rescue the blood–brain barrier integrity.

The involvement of ERK in brain damage has been reported. In primary rat cortical cultures, treatment with PD98059, which inhibits MAPK/ERK 1/2, the upstream activator of ERK, significantly increased cell survival in vitro [34]. Blocking activation of extracellular signal-regulated kinase (ERK) with the MEK inhibitor U0126 mitigates brain damage in rodent models of ischemic stroke. Together with our data, it is indicated that critical perturbations in MAPK pathways mediate cerebral damage after acute injury, and it is further suggested that ERK is a novel therapeutic target in brain injury.

## Conclusions

Our study showed that after ischemia induction, the phosphorylation of Cx43 increased. The Cx40/Cx43 and Cx40/phosphorylated Cx43 heteromeric complex also increased and the increased heteromeric complex contributes to brain damage. Cx43 is the substrate of ERK and inhibition of ERK results in inhibition of Cx43 phosphorylation. Inhibition of ERK protects blood–brain barrier integrity and prevents brain damage, suggesting ERK as a potential therapeutic target in brain injury.

## Abbreviations

Cx40: connexin40
BBB: blood–brain barrier
CNS: central nervous system
TBI: traumatic brain injury
GS-Rb1: ginsenoside Rb1
